# The Fragile Patient: Considerations in the Management of Invasive Mould Infections (IMIs) in India

**DOI:** 10.7759/cureus.38085

**Published:** 2023-04-24

**Authors:** Prithwijit Kundu, Neha Gupta, Nitin Sood

**Affiliations:** 1 Medical Affairs, Pfizer, Mumbai, IND; 2 Internal Medicine, Fortis Memorial Research Institute, Gurugram, IND; 3 Internal Medicine, Medanta - The Medicity, Gurugram, IND; 4 Hematology and Oncology/Stem Cell Transplant, Cancer Institute, Medanta - The Medicity, Gurugram, IND

**Keywords:** isavuconazole, aspergillosis, mucormycosis, invasive mold infection, mycoses, immunocompromised, fragile

## Abstract

Invasive mould infections (IMIs), which are mostly caused by *Aspergillus spp.* and *Mucormycetes*, are opportunistic infections that impose a substantial threat to patients who are considered to be ‘fragile’. There is no fixed definition for fragile patients; however, patients with cancer or acquired immunodeficiency syndrome (AIDS), patients who have undergone organ transplants, and patients being treated in the intensive care units (ICUs) were considered fragile. Management of IMIs in fragile patients is challenging, owing to their compromised immune status. The diagnostic challenges associated with IMIs due to insufficient sensitivity and specificity of the current diagnostic tests lead to delayed treatment. A widening demographic of at-risk patients and a broadening spectrum of pathogenic fungi have added to the challenges to ascertain a definite diagnosis. A recent surge of mucormycosis associated with SARS-CoV-2 infections and the resultant steroid usage has been reported. Liposomal amphotericin B (L-AmB) is the mainstay for treating mucormycosis while voriconazole has displaced amphotericin B as the mainstay for treating *Aspergillus* infection due to its better response, improved survival, and fewer severe side effects. The selection of antifungal treatment has to be subjected to more scrutiny in fragile patients owing to their comorbidities, organ impairment, and multiple ongoing treatment modalities. Isavuconazole has been documented to have a better safety profile, stable pharmacokinetics, fewer drug-drug interactions, and a broad spectrum of coverage. Isavuconazole has thus found its place in the recommendations and can be considered a suitable option for treating fragile patients with IMIs. In this review, the authors have critically appraised the challenges in ascertaining an accurate diagnosis and current management considerations and suggested an evidence-based approach to managing IMIs in fragile patients.

## Introduction and background

Invasive fungal infections (IFIs) increase morbidity and mortality in the vulnerable population and impose a substantial burden on critical care units. Invasive mould infections (IMIs) are mostly caused by *Aspergillus spp.* and *Mucormycetes*. Each of these two infections requires a different diagnostic and management approach [1,2]. Early diagnosis and newer antifungal drugs have managed to curb mortality in immunocompromised patients with IMIs [3]. The challenges currently associated with antifungal pharmacotherapy include a surge in resistance to antifungals, breakthrough fungal infections with inherently resistant fungi, potentially life-threatening adverse effects, and drug-drug interactions, especially with extended therapy [3]. The demographics of cancer patients, patients with acquired immunodeficiency syndrome (AIDS), patients who have undergone organ transplants, and patients being treated in the intensive care units (ICUs) can be considered to be ‘fragile’ patients [4]. The risk of getting an invasive mycological infection is higher in fragile patients. The antifungal options are limited due to the obvious demand for higher safety [4]. Although there is no fixed definition, these patients can be considered to be fragile because of their compromised immunity owing to the primary diagnosis, potential drug-drug interactions due to multiple ongoing pharmacotherapies, adverse drug reactions from the pharmacotherapy of the primary disease, existing organ dysfunction (e.g., hepatic and renal), and added toxicity of antimicrobials.

Thus, there is a distinct challenge in treating fragile patients. In this review, the authors have critically appraised the changes in the epidemiology of fungal infections, the challenges in ascertaining an accurate diagnosis, and current management considerations, and suggested an evidence-based approach to managing IMIs in fragile patients.

## Review

Epidemiology of IMIs in India

Globally, each year about 250,000 cases of invasive aspergillosis (IA) and about 10,000 cases of mucormycosis are reported. There is limited information on the global incidence of these infections due to the absence of national surveillance systems. Additionally, there is no compulsion to report fungal diseases, and the clinicians might miss the signs and diagnostic tests may not be conclusive [5]. Considering these clinical challenges, there is a possibility of under-reporting of IFIs [5,6].

The prevalence of mucormycosis in Asia is considerably higher as compared to Europe (i.e., 14 per 100,000 people vs. <0.6 per 100,000 people) and has been increasing over the recent decades [5,7,8]. There has been a recent surge of mucormycosis associated with SARS-CoV-2 infections and the resultant steroid usage [9]. There is a paucity of prevalence data specifically for IA from India. The available epidemiological data from India are summarised in Table 1.

**Table 1:** Epidemiology of invasive mould infections (IMIs) CAM, COVID-19-associated mucormycosis; ICU, intensive care unit; ROCM, rhino-orbital-cerebral mucormycosis.

Sr. No.	Reference	Region/country	Type of study	Population	Type of infection	Study period	Incidence/prevalence	Highlights
1.	Patel et al. (2021) [10]	India	Multicentre, retrospective	Confirmed mucormycosis cases among patients with and without COVID-19	Mucormycosis	September 2020 – December 2020	287 cases	187 (65.2%) had CAM. CAM prevalence was 0.27% among hospitalised COVID-19 patients
2.	Sen et al. (2021) [11]	India	Retrospective, multicentric, non-interventional, observational study	Patients with rhino-orbital-cerebral mucormycosis (ROCM)	ROCM	January 2020 – May 2021	2,826 cases	78% of patients had diabetes mellitus with a mean HbA1c of 9.8 (measured in 466 patients) - 41% of diabetic patients had uncontrolled diabetes
3.	Patel et al. (2020) [12]	India	Prospective observational study	Patients with proven mucormycosis	Mucormycosis	January 2016 – September 2017	485 cases	73.5% of patients had diabetes mellitus
4.	Priya et al. (2020)[13]	India	Single-centre, retrospective	Patients diagnosed with proven and probable mucormycosis	Mucormycosis	October 2015 – October 2019	38 cases	77% of patients had diabetes mellitus
5.	Chakrabarti et al. (2019) [14]	India	Prospective observational study	ICU patients	Invasive mould infections	April 2016 – September 2017	Prevalence: 9.5 cases per 1,000 ICU admissions	*Aspergillus* isolated in 82.1%, *Mucorales* isolated in 14.4%
6.	Chander et al. (2018) [15]	India	Prospective observational study	Patients with suspected mucormycosis admitted to the hospital	Mucormycosis	January 2010 – December 2014	82 cases	62.2% of patients had diabetes mellitus
7.	Rotjanapan et al. (2018) [16]	Thailand, Taiwan, Singapore, China, India	Retrospective study	Data were collected from discharge/death diagnosis, microbiology/mycology laboratory records, and histopathology laboratory records	Invasive mould infections	January 2012 – December 2012	155 cases. Incidence: Singapore - 0.0444 per 1,000 patient days; Thailand - 0.2621 per 1,000 patient days; China - 0.0242 per 1,000 patient days; India - 0.0308 per 1,000 patient days; Taiwan - 0.0531 per 1,000 patient days	30.9% of patients had diabetes mellitus 3.2% had undergone a solid organ transplant
8.	Patel et al. (2017) [17]	India	Retrospective observational chart review	Patients diagnosed with proven and probable mucormycosis	Mucormycosis	January 2013 – May 2015	24 cases	55.6% of patients had diabetes, 11.1% had undergone a solid organ transplant

Diagnostic challenges

Imaging using computed tomography (CT), microbiological tools, and histopathology are the pillars of the diagnostic workup of IMI. Cultures of secretions retrieved from the lower respiratory tract using bronchoscopy or bronchoalveolar lavage fluid (BAL) and galactomannan and (1,3)-β-D-glucan are key diagnostic tools to identify pulmonary fungal infections and aspergillosis, respectively. However, establishing a diagnosis of IMI is difficult due to the insufficient sensitivity and specificity of the current diagnostic tests. A widening in the spectrum of pathogens causing IMI and in the demographics of immunocompromised patients has added to the challenges of a proper diagnosis. Although advances in polymerase chain reaction (PCR) techniques have eased the diagnostic challenges for IA and invasive mucormycosis (IM) to a certain degree, it is not available at all medical facilities [18]. Since the currently available diagnostic tests provide insufficient sensitivity and specificity, the optimal approach is to combine multiple diagnostic strategies, including imaging, fungal biomarkers (galactomannan and (1,3)-β-D-glucan), and molecular tools [18,19].

The Challenge of Differentiating Invasive Aspergillosis From Mucormycosis

Mucormycosis and IA have common clinical and radiological signs. Lesions (such as halo sign, reverse halo sign (RHS), nodules, cavities, wedge-shaped effusions, and pleural effusions), which are observed with pulmonary mucormycosis, are also observed with *Aspergillus spp. *and *Pseudomonas* *aeruginosa *infections [20]. Hence, a high index of suspicion along with host factors and the presence of clinical signs are required to make early identification of mucormycosis [20,21]. Mucormycosis should be considered when there is a history of prior voriconazole use or an RHS (in lung parenchyma) on high-resolution computed tomography [20,22]. Multiple (≥10) nodules along with pleural effusion picked up on a radiograph indicate pulmonary mucormycosis [23]. Rhinocerebral mucormycosis can be diagnosed in diabetic patients using an algorithm enlisting the 'red flags' such as diplopia, periorbital swelling, cranial nerve palsy, sinus pain, orbital apex syndrome, proptosis, and palate ulcers [24]. Another helpful investigative approach for ascertaining a diagnosis is fine needle aspiration, guided by endobronchial ultrasound [21].

Management considerations

The modern antifungals for the treatment of mould infections consist of three chief classes: triazoles, echinocandins, and amphotericin B (AmB). These three classes are individually limited by their spectrum [3].

The general treatment principles in the management of IMIs in immunocompromised patients, which can be considered while developing an effective personalised treatment strategy, are as follows: knowing the spectrum of activity of commonly used antifungals; understanding epidemiology of IMIs; knowing the pathogenesis as well as natural history of the mould infection to enable effective use of therapy in high-risk patients; knowledge of pharmacokinetics and pharmacodynamics of the antifungals; understanding the adverse effects associated with antifungals; knowing importance of early diagnosis to warrant sufficient treatment response; understanding that prognosis is severely dependent on the degree and course of immunosuppression in the patient; understanding that acute versus chronic states of treatment can influence selection of antifungal agent; implementation of multidisciplinary approach; and acknowledging that evidence-based guidelines provide a starting point and not a road-map for managing patients [3].

The approved treatment modalities along with their approved indications have been summarised in Table 2.

**Table 2:** Antifungal therapies indicated for the treatment and prophylaxis of invasive aspergillosis and mucormycosis EU, European Union; GVHD, graft versus host disease; HSCT, haematopoietic stem cell transplantation; IV, intravenous; US, United States. Source: Data from the European Medicines Agency, 2018 [25], Food and Drug Administration, 2015 [26], EMC, 2017 [40], US Department of Health and Human Services – AIDS info, 2017 [41], EMC, 2017 [42], US Department of Health and Human Services – AIDS info, 2017 [43], EMC, 2017 [28], Food and Drug Administration, 2008 [29], EMC, 2016 [32], Food and Drug Administration, 2005 [33], EMC, 2017 [44], Food and Drug Administration, 2012 [45], European Medicines Agency, 2018 [36], Food and Drug Administration, 2015 [35], European Medicines Agency, 2018 [37], and Food and Drug Administration, 2015 [38].

Treatment			Relevant indications in Europe, the US, & India	
Isavuconazole [25-27]				
Europe	Treatment of invasive aspergillosis. Treatment of mucormycosis in patients for whom amphotericin B is inappropriate			
US	Treatment of invasive aspergillosis. Treatment of invasive mucormycosis			
India	Treatment of invasive aspergillosis. Treatment of invasive mucormycosis			
Liposomal amphotericin B (L-AMB) [28-31]				
Europe		Treatment of severe systemic and/or deep mycoses. Empirical treatment of presumed invasive fungal infections in febrile neutropenic patients. Indication mentions that L-AMB has been used to treat both aspergillosis and mucormycosis; however, it should not be used to treat common clinically inapparent forms of fungal disease, which show only positive skin or serologic tests		
US		Empirical therapy for presumed fungal infection in febrile, neutropenic patients. Treatment of patients with *Aspergillus* species refractory to amphotericin B deoxycholate, or in patients where renal impairment or unacceptable toxicity precludes the use of amphotericin B deoxycholate		
India		Prophylactic, empirical or treatment of disseminated and invasive systemic fungal infections		
Caspofungin [32-34]				
Europe		Empirical treatment of presumed invasive fungal infections, such as *Aspergillus* in febrile neutropenic patients		
US		Empirical therapy for presumed fungal infections in febrile, neutropenic patients. Treatment of invasive aspergillosis in patients who are refractory to or intolerant of other therapies (i.e., amphotericin B, lipid formulations of amphotericin B, and/or itraconazole)		
India		Empirical therapy for presumed fungal infections in febrile neutropenic patients. Treatment of invasive *Aspergillosis *in patients who are refractory to or intolerant of other therapies (i.e., amphotericin B, lipid formulations of amphotericin B, and/or itraconazole)		
Posaconazole [34-36]				
Europe		Invasive aspergillosis in patients with a disease that is refractory to amphotericin B or itraconazole or in patients who are intolerant of these medicinal products		
US		No treatment indication. Only prophylaxis of invasive *Aspergillus* infections in patients who are at high risk of developing this infection due to being severely immunocompromised, such as HSCT recipients with GVHD or those with haematologic malignancies with prolonged neutropenia from chemotherapy		
India		Mucormycosis in patients with disease refractory to other therapy, or patients who are intolerant of other therapy. For the treatment of oropharyngeal candidiasis, including oropharyngeal candidiasis refractory to itraconazole and/or fluconazole. For prophylaxis of invasive *Aspergillus* and *Candida *infections in patients, 13 years of age and older, who are at high risk		
Voriconazole [37-39]				
Europe		Treatment of invasive aspergillosis		
US		Treatment of invasive aspergillosis		
India		Treatment of invasive aspergillosis		

Immunocompromised Patients

*Aspergillus spp. *and *Mucorales* are opportunistic fungi that frequently cause menace for immunocompromised patients, including organ transplant patients, haemato-oncological patients, and immunodeficiency syndrome patients [46].

Voriconazole has displaced AmB as the mainstay for treating *Aspergillus* infection in immunocompromised patients [46]. Liposomal amphotericin B (L-AmB) is recommended as the first-line agent for treating mucormycosis by the European Conference on Infections in Leukaemia (ECIL, 2017) and the European Confederation of Medical Mycology (ECMM, 2019) [46,47]. The ECMM 2019 guidelines strongly recommend high-dose L-AmB as the first-line treatment for mucormycosis in the general population. Isavuconazole and posaconazole (intravenous or delayed-release tablets) have moderate strength of recommendation as the first-line treatment [48].

Solid Organ Transplantation

IFIs increase mortality and morbidity in patients undergoing solid organ transplants (SOTs). SOT is imperative for patients with end-stage organ failure. Hence, the prevention and treatment of fungal infections are crucial. Voriconazole is the suggested first-line therapy for IA in SOT patients. Other agents for IA include L-AmB, AmB lipid complex, isavuconazole, caspofungin, and micafungin. Echinocandins have only static activity against aspergillosis as they act against the growing fungal hyphae. A combination of voriconazole and echinocandin is advised to be reserved as salvage therapy [49]. Mucormycosis infection is rare in SOT patients but with a fatality rate of up to 60%. Managing mucormycosis often involves surgical excision or debridement of the necrotic area along with intravenous antifungals. L-AmB is recommended as induction therapy and isavuconazole as the first-line agent. Posaconazole can be given as salvage therapy to patients not unresponsive to AmB. Isavuconazole is recommended for maintenance and also as salvage therapy in SOT patients [49].

Haematologic Malignancy

IFI adds to the morbidity and mortality in patients with haematologic malignancies and patients going through haematopoietic cell transplant (HCT) [50]. Clinicians might need to consider the possibility of IA in such patients with a fever of more than three to four days. It is advised to start empiric antifungals after 96 hours of fever that persists even after empirical antibiotic treatment [51]. Posaconazole is recommended for prophylactic use in cases with prolonged neutropenia due to chemotherapy for acute myeloid leukaemia (AML) or myelodysplastic syndrome (MDS) and in HCT recipients requiring augmented immunosuppression for graft vs. host disease (GVHD) [50]. The initial treatment of invasive pulmonary aspergillosis (IPA) can be done with voriconazole or isavuconazole, except in cases where it is a breakthrough infection due to azole prophylaxis. IA sinusitis should be treated with surgical debridement combined with systemic antifungals. Initial treatment with triazole may reduce the six-week mortality rate [51]. IA of the central nervous system (CNS) is mitigated with surgical procedures and voriconazole [51]. ECIL-6 strongly recommend voriconazole and isavuconazole for treating IA in leukaemia patients and patients undergoing haematopoietic stem cell transplant [52].

For treating mucormycosis in stem cell transplant recipients and haematologic malignancy patients, L-AmB is the preferred antifungal that can be combined with an echinocandin [51]. Isavuconazole is advised in patients who cannot endure AmB. Voriconazole, however, is ineffective [51]. ECIL-6 recommends a multidisciplinary approach, including antifungal, surgery, and controlling underlying conditions in leukaemia patients and patients undergoing haematopoietic stem cell transplant [52].

Critically Ill Patients in ICU

Opportunistic fungi often take advantage of the immunocompromised state of critically ill patients admitted to the ICU. In response to sepsis, a biphasic immunological pattern is observed. It consists of an early hyperinflammatory phase trailed by an anti-inflammatory response, causing a hypo-inflammatory state. This is known as compensatory anti-inflammatory response syndrome (CARS or immunoparalysis) [53]. Treatment of IA with first-line therapy (voriconazole or isavuconazole), at an early stage when the infection is suspected, improves outcomes and mortality. For managing infection with *Mucorales*, a combination of correcting the underlying conditions where feasible, surgical resection when possible, and antifungal therapy is required [1].

Diabetic Patients

Mucormycosis is becoming a progressively prevalent infection in diabetes mellitus patients whose blood glucose levels are not well managed. Hyperglycaemia impairs acquired and innate immunity and increases the chances of getting IA [1]. Diabetes mellitus is the chief risk factor for mucormycosis in India. The prevalence of mucormycosis in India is hence much higher (14 per 100,000 population). The prevalence rate in the United States and Europe ranges from 0.01 to 0.2 per 100,000 population, which is lower compared to Indian estimates [54,55]. In patients with uncontrolled diabetes and suspected mucormycosis, rapid correction of metabolic aberrations is obligatory along with pharmacotherapy with antifungal agents [55]. L-AmB has a broad spectrum and is efficacious for most fungal infections. It remains the first-line agent for treating mucormycosis. However, the formulation contains about 900 mg of sucrose per vial, which may prove detrimental in hyperglycaemic patients. Isavuconazole is the recommended second-line agent along with posaconazole in patients who cannot tolerate L-AmB [47,56].

COVID-19-Associated Fungal Infections

The ongoing SARS-CoV-2 or coronavirus disease 2019 (COVID-19) pandemic has given rise to secondary infections known as COVID-19-associated pulmonary aspergillosis (CAPA) and COVID-19-associated mucormycosis (CAM) [57,58]. India has reported a significant burden of IM as a fatal complication of COVID-19 [59]. Diagnosis of CAPA and CAM is especially difficult considering the fragile state of the patients [57]. For diagnosing CAPA, the conventionally used procedures for IA are at a disadvantage because they either are unsuitable for testing the lower respiratory tract (e.g., testing sputum, non-bronchoscopic lavage, and tracheal aspirate) or they risk contamination (e.g., bronchoscopy with BAL) by SARS-CoV-2. BAL testing is preferable for diagnosing IPA in COVID-19 patients [58]. For ascertaining mucormycosis, diagnostic procedures employed are biopsy or mycological examination with potassium hydroxide (KOH) mount and calcofluor stain. A biopsy is the mainstay of diagnosis and the benefits of performing the test outweigh the risk, even in a ‘difficult to access’ location or in the presence of coagulopathy [59].

The 2020 ECMM/International Society for Human and Animal Mycology (ISHAM) consensus criteria for research and clinical guidance recommends voriconazole or isavuconazole as first-line agents for the management of CAPA. For azole-resistant variants, voriconazole or isavuconazole plus echinocandin is recommended for suspected CAPA and L-AmB for suspected or proven CAPA [60]. AmB is the preferred antifungal for treating CAM. Due to possible renal impairment, isavuconazole and posaconazole may be advised. Adjuvant therapy with caspofungin, statins, aspirin, and hyperbaric oxygen may be considered on a need basis [59].

COVID-19-Associated Mucormycosis in India

A multicentre study was conducted in India from September to December 2020, across 16 healthcare centres. Among them, seven centres reported 112 cases of mucormycosis in 2019 and 231 cases in 2020, of which 139 (60.2%) were CAM. A surge in CAM cases is thus evident. From the 16 centres, during the study period, 287 cases of mucormycosis were reported, of which 187 (65.2%) had CAM. The overall prevalence of CAM is estimated to be 0.27%. A higher proportion of cases of CAM have been observed in the older population (mean age of the study population: 56.9 years) and the male gender (80.2%). Uncontrolled diabetes was found to be the common underlying issue for both CAM and non-CAM patients. Interestingly, newly detected diabetes mellitus was more frequently noted in CAM patients as compared to non-CAM (20.9% vs. 10%). As compared to non-CAM patients (84%), the use of L-AmB was lower in CAM patients (72.7%). CAM patients were more frequently treated with isavuconazole and posaconazole. The mortality rate among the two groups was found to be similar, i.e., 38.3% at six weeks. Hypoxemia due to COVID-19 and inappropriate glucocorticoid use were determined to be the causative factors for late CAM [10]. A case-control study conducted across 25 hospitals in India during January-June 2021 reported 1,733 cases of CAM with a mortality rate of 32.2%. The study concluded that the unmonitored use of medications like glucocorticoids and zinc supplements in addition to host factors (renal transplantation, diabetes mellitus, and elevated C-reactive protein) was associated with CAM [61]. Another retrospective, observational study of patients with COVID-19-associated rhino-orbital-cerebral mucormycosis (ROCM) including 2,826 patients ascertained the use of corticosteroids and diabetes mellitus as predisposing factors. The authors also suggested that treatment with antifungals can be initiated empirically upon suspicion due to clinical or clinical-radiological correlation in a symptomatic patient with COVID-19 [62].

Pharmacokinetic considerations

Hepatic and Renal Dysfunction

The therapeutic decisions for treating mould infections should be done in consideration of the impairment of hepatic and/or renal function and the potential drug-drug interactions [63]. A summary of the required dose adjustments for various antifungals is present in Table 3 [26,27,29,32,33,35,40,41,62,64].

**Table 3:** Dose adjustments for antifungals in patients with hepatic and/or renal dysfunction * Consider hepatotoxicity. # Consider nephrotoxicity. b.i.d, twice daily; GFR, glomerular filtration rate; IV, intravenous; L-AmB, liposomal amphotericin B; q.i.d, four times daily; SBECD, sulfobutylether-β-cyclodextrin; SmPC, summary of product information; TDM, therapeutic drug monitoring; t.i.d, thrice daily.

Sr. No.	Antifungal agent	Route of administration and dose	Required dose adjustment	
			Hepatic impairment	Renal impairment
1.	L-AmB	5 mg/kg	No dose adjustment needed*	No dose adjustment needed^#^
2.	Amphotericin B deoxycholate	1 mg/kg	No dose adjustment needed*^#^	Contraindicated in reversible renal impairment
3.	Voriconazole	IV: loading dose 6 mg/kg b.i.d on day 1; maintenance dose 4 mg/kg b.i.d. Oral: loading dose 400 mg b.i.d on first day; maintenance dose 200 mg b.i.d	For mild to moderate hepatic impairment 50% dose reduction with TDM	Standard dose, consider SBECD accumulation during IV infusion
4.	Isavuconazole	IV: loading dose 200 mg t.i.d on day 1 and day 2; maintenance dose 200 mg once daily. Oral: loading dose 200 mg t.i.d on day 1 and day 2; maintenance dose 200 mg once daily	Standard dose	Mild to moderate, enhanced levels, no dose reduction recommended
5.	Posaconazole (oral suspension)	Therapeutic dose: 200 mg q.i.d or 400 mg b.i.d. Prophylaxis dose: 200 mg t.i.d	No dose adjustment	No dose adjustment
6.	Posaconazole (tablet)	Loading dose: 300 mg b.i.d on day 1. Maintenance dose: 300 mg once daily	No dose adjustment	No dose adjustment
7.	Posaconazole (IV)	Loading dose: 300 mg b.i.d on day 1. Maintenance dose: 300 mg once daily	No dose adjustment	GFR < 50 mL/min: avoid because of cyclodextrin accumulation
8.	Itraconazole	Loading dose: 200 mg TDS for 3 days. Maintenance dose: 200 mg b.i.d	Consider dose reduction, TDM	No dose reduction, enhanced dose during continuous renal replacement therapy
9.	Caspofungin	Loading dose: 70 mg once daily. Maintenance dose: 50 mg once daily (70 mg if body weight is >80 kg)	Enhanced exposure in moderate hepatic impairment, dose reduction	No dose adjustment
10.	Micafungin	50 mg once daily for prophylaxis	Slightly lowered concentrations, contra-indicated in European SmPC	No dose adjustment

Drug-Drug Interactions

Voriconazole has a high risk of drug-drug interactions, especially in the Asian population due to the substantial proportion of slow metabolizers. Its concomitant use with immunosuppressants, including sirolimus, tacrolimus (nephrotoxic drug), and cyclosporine, is advised to be monitored and dose adjustments should be done as required since voriconazole causes a significant increase in their plasma concentrations. Proton pump inhibitors are competitive inhibitors of voriconazole metabolism and hence can increase voriconazole plasma concentrations, leading to concentrations outside the therapeutic range, which can be associated with either impaired treatment of IA or increased toxicity for the patient [62,65,66].

Posaconazole is a potent inhibitor of CYP3A4 and can cause significant drug-drug interactions with other medications metabolized by this enzyme [67]. Co-administration with terfenadine, astemizole, cisapride, pimozide, halofantrine, and quinidine may increase plasma concentrations of these medical products [35]. This can lead to QTc prolongation, which has been linked with cardiovascular events such as torsades de pointes [68]. Posaconazole’s increased risk of drug-drug interactions, coupled with its non-linear pharmacokinetics, which leads to a large inter- and intra-individual variation in bioavailability, results in the need for therapeutic drug monitoring (TDM) when using oral suspension [69-71]. Azole levels decrease significantly with rifampicin [26,38,45,72].

Isavuconazole is a moderate CYP3A4 inhibitor and does not inhibit either CYP2C9 or CYP2C19. The current US and European Union prescribing information does not recommend dose adjustment with concomitant administration of isavuconazole and ciclosporin, tacrolimus, or sirolimus. However, monitoring the drug concentrations of the immunosuppressive agents with dose adjustments is advised as required [73]. Triazoles are associated with QTc prolongation. Isavuconazole, unlike other triazoles, causes a dose-dependent QTc interval shortening, whose clinical significance is unknown. The safety profile of isavuconazole is more favourable as compared to other azoles [74]. The SECURE trial reported less drug-associated hepatotoxicity with isavuconazole when compared to voriconazole [75].

Therapeutic Drug Monitoring Considerations

TDM is a useful tool to monitor the safety and efficacy of antifungals with a narrow therapeutic window and unpredictable pharmacokinetics with a well-defined exposure activity relationship. Considering that the patients at risk of systemic fungal infections include fragile and immunocompromised patients dealing with a myriad of other health concerns, TDM helps optimise the management approach [71,76]. The antifungal agents that are recommended for a routine TDM are itraconazole, voriconazole, posaconazole, and flucytosine. Flucytosine is under the TDM scanner due to its toxic potential and interpatient variability concerning kidney function. Voriconazole displays significant interpatient variability in pharmacokinetics. Hence, TDM helps in ensuring therapeutic concentration is achieved in these patients. Posaconazole’s TDM is dependent on the type of formulation in question. The delayed-release tablets and intravenous formulations have a relatively stable pharmacokinetic profile. TDM is critical when the suspension is used to monitor therapeutic levels. TDM is also needed when potential drug-drug interactions are identified [76]. Isavuconazole has a dose-dependent pharmacokinetic profile with minimal variability. The intra-subject variability is also minimal. Hence, TDM is not recommended for isavuconazole [74]. Although TDM could be beneficial in monitoring and clinical evaluation of isavuconazole-treated individuals for situations like unforeseen toxicity, treatment failure, and pharmacokinetic drug-drug interactions. TDM will also prove useful if pathogens with elevated minimum inhibitory concentration (MIC) or infections at sanctuary sites (e.g., CNS) are being treated with isavuconazole. A plasma trough of 2-3 mg/L range (mean concentration range from phase II/III clinical studies) after day five (including loading doses) indicates an acceptable drug exposure, in case precise therapeutic targets are missing [77].

Pharmacodynamic considerations

*Aspergillus fumigatus* is the most common causative agent for IA globally. But, in Asian, African, and Middle Eastern regions, *Aspergillus* *flavus* is known to be the predominant causative agent. Approximately 10% of global bronchopulmonary aspergillosis cases are due to *A. flavus* [14]. It is known to be inherently resistant to polyenes while triazole resistance is occasionally observed in *A. fumigatus*.* A. flavus* is resistant to AmB probably due to higher ergosterol levels and a rise in enzymatic activity of the peroxidase and superoxide dismutase, with decreased lipid peroxidation. The definite mechanism for the resistance is undetermined. The MIC of AmB for *A. flavus* is high. Voriconazole and isavuconazole are recommended drugs of choice to treat IA. Echinocandins may be added to make a combination therapy if the situation demands. AmB preparations are advised to be avoided [14]. Isavuconazole has been reported to have favourable* in vitro *antifungal activity against clinically significant *Aspergillus* and *Mucorales* isolates. Its MIC range against *Mucor spp. *is <0.015 to >8 µg/mL and its minimum fungicidal concentration (MFC) range is 2 to >16 µg/mL [78]. The European Committee on Antimicrobial Susceptibility Testing (EUCAST)-issued clinical MIC breakpoints for isavuconazole for *A. fumigatus* are ≤1 µg/mL for susceptible and ≤1 µg/mL for resistant isolates [78].

Evidence-based approach in managing IMI in the fragile patient

Various guidelines have been developed to provide recommendations for the treatment of IMIs in different populations. These guidelines use graded strength of evidence and different levels of quality of evidence to make the recommendations [48,52,77,79].

The ECIL guidelines published in 2017 are formulated by the European Hematology Association for patients with haematologic malignancies or haematopoietic stem cell transplantation recipients. They comprise recommendations for diagnosis, prophylaxis, and preventive or targeted therapy for various types of fungal infections in such patients (Table 4) [52].

**Table 4:** ECIL-6, ESCMID-ECMM-ERS#, and ECMM/ISHAM consensus on CAPA guidelines * Monitoring of serum levels is indicated. In the absence of sufficient data for first-line monotherapy, anidulafungin, micafungin, and posaconazole have not been graded. ^#^ Populations: Neutropenia (non-Allo-HSCT recipients), Allo-HSCT (during neutropenia) and Allo-HSCT (w/o neutropenia), or other nonneutropenic patients. ^¶^ Therapeutic drug monitoring is required. ^§ ^Continuous monitoring of renal function is recommended. ^† ^The optimal duration is unknown, but the expert panel suggests six to 12 weeks as a treatment course. In immunocompromised patients (e.g., with haematological malignancy or receiving immunosuppressive therapy), longer treatment might be necessary. ^‡^ Salvage therapy: caspofungin 70 mg loading dose on the first day followed by 50 mg/day. If body weight is more than 80 kg, then 70 mg loading dose on the first day followed by 70 mg/day. AmB, amphotericin B; Allo-HSCT, allogeneic haematopoietic stem cell transplantation; BAL, bronchoalveolar lavage; bid, two times daily; CAPA, COVID-19-associated pulmonary aspergillosis; d-AmB, amphotericin B deoxycholate; ECIL, European Conference on Infections in Leukaemia; ECMM/ISHAM, European Confederation of Medical Mycology and International Society For Human and Animal Mycology; ESCMID-ECMM-ERS, European Society for Clinical Microbiology and Infectious Diseases-European Confederation of Medical Mycology-European Respiratory Society; GM, galactomannan; IA, invasive aspergillosis; IDSA, Infectious Diseases Society of America; IV, intravenous; L-AmB, liposomal amphotericin B; PCR, polymerase chain reaction; qd, one time daily; QoE, quality of evidence; SoR, strength of recommendation; TDM, therapeutic drug monitoring; tid, three-times daily dosing. Source: Tissot et al. (2017) [52], Ullmann et al. (2018) [77], Patterson et al. (2016) [79], and Koehler et al. (2021) [60].

	SoR	QoE		Comments
ECIL-6				
Voriconazole*	A	I		Daily dose: 2 x 6 mg/kg on day 1 then 2 x 4 mg/kg (initiation with oral therapy: C III)
Isavuconazole	A	I		As effective as voriconazole and better tolerated
Liposomal AmB	B	I		Daily dose: 3 mg/kg
AmB lipid complex	B	II		Daily dose: 5 mg/kg
AmB colloidal dispersion	C	I		Not more effective than d-AmB but less nephrotoxic
Caspofungin	C	II		
Itraconazole	C	III		
Recommendation against the use of d-AmB deoxycholate	A	I		Less effective and more toxic
ESCMID-ECMM-ERS				
Isavuconazole 200 mg IV tid on day 1 and 2, then 200 mg qd oral	A	I		D III, if mould active azole prophylaxis; has fewer adverse effects than voriconazole
Voriconazole 2 x 6 mg/kg IV on day 1, then 2 x 4 mg/kg IV (oral 200 mg bid)	A	I		C III for start with oral; D III, if prior mould active azole prophylaxis; TDM
L-AmB 3 mg/kg	B	II		-
Caspofungin 70 mg qd on day 1, followed by 50 mg qd (if body weight <80 kg)	C	II		-
Itraconazole 200 mg q12 h IV on day 1, then 200 mg/qd	C	III		D III for start with oral, TDM D III, if mould active azole prophylaxis
AmB lipid complex 5 mg/kg	C	III		-
Micafungin 100 mg	C	III		-
AmB colloidal dispersion 4-6 mg/kg	D	I		-
Conventional AmB 1-1.5 mg/kg	D	I		-
Other combinations	D	III		Efficacy unproven
IDSA				
Primary: voriconazole (6 mg/kg IV every 12 hours for 1 day, followed by 4 mg/kg IV every 12 hours; oral therapy can be used at 200 mg every 12 hours)		Alternative Primary: AmB (3-5 mg/kg/day IV) 200 mg every 8 hours for 6 doses, then 200 mg daily. Salvage: (70 mg/day IV × 1, then 50 mg/day IV thereafter) (100-150 mg/day IV), Posaconazole (oral suspension: 200 mg tid; tablet: 300 mg bid on day 1, then 300 mg daily, IV: 300 mg bid on day 1, then 300 mg daily		
ECMM and ISHAM CAPA guidelines				
Azole sensitive: First-line voriconazole^¶^ (day 1: 2 x 6 mg/kg per day; day 2 to^†^: 2 x4 mg/kg per day). Isavuconazole (days 1-2: 3 x 200 mg per day; day 3 to^†^: 1 x 200 mg per day). Second-line liposomal amphotericin B^‡^ (3 mg/kg per day)			Azole resistant: Suspected voriconazole plus echinocandin OR isavuconazole plus echinocandin. Suspected or proved liposomal amphotericin B^§^ (3 mg/kg per day)	

The focus of the European Society for Clinical Microbiology and Infectious Diseases, the European Confederation of Medical Mycology and the European Respiratory Society (ESCMID-ECMM-ERS) Joint Clinical Guidelines on targeted first-line therapy for pulmonary diseases is outlined in Table 4 [77]. Similar to ECIL-6 guidelines, ESCMID-ECMM-ERS guidelines also recommend use of isavuconazole at the same level as voriconazole and better than amphotericin B preparation. The guideline also mentions that isavuconazole has fewer adverse effects as compared to voriconazole.

The Infectious Diseases Society of America (IDSA) periodically releases guidelines on the diagnosis and management of aspergillosis. The latest guidelines published in 2016 are summarised in Table 4 [79]. Isavuconazole was approved by the Food and Drug Administration (FDA) in 2015. Despite being a newcomer to the market, IDSA recommended the use of isavuconazole as a primary treatment in 2016, suggesting its impact as an antifungal agent and an effective alternative to voriconazole.

Considering the current challenges faced by the medical fraternity due to COVID-19 and associated fungal infections, the ECMM and the ISHAM have published updated recommendations for the diagnosis and treatment of patients with CAPA (summarised in Table 4) [60].

Isavuconazole has been given the same recommendation level and strength as voriconazole. But it is well noted that it is highlighted to be better tolerated as compared to voriconazole, which is tagged for TDM. Isavuconazole also has a better recommendation profile as compared to AmB preparations.

Guidelines on Invasive Mucormycosis

**Figure 1:**
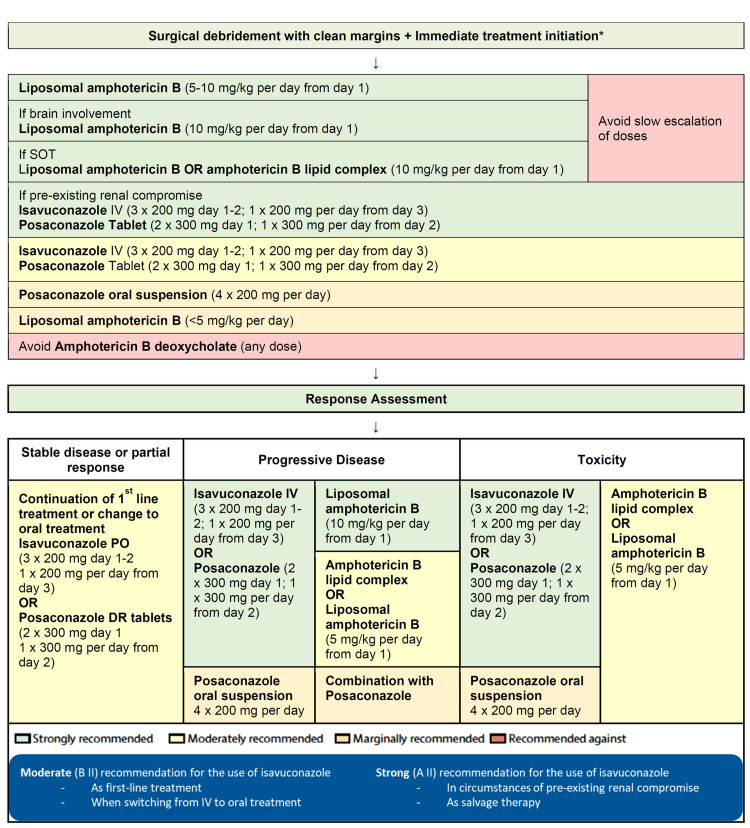
ECMM-MSG-ERC guidelines: optimal treatment pathways for mucormycosis in adults * For three purposes: (a) disease control, (b) histopathology, and (c) microbiological diagnostics. ECMM-MSG-ERC: European Confederation of Medical Mycology-Mycoses Study Group Education and Research Consortium; IV, intravenous; PO, per os (taken orally); SOT, solid organ transplantation; DR, delayed release. Adapted from: Cornely et al. (2019) [48].

The 2019 global guidelines for the diagnosis and management of mucormycosis were published based on an initiative of the ECMM in cooperation with the Mycoses Study Group Education and Research Consortium (MSG-ERC) [48]. The management recommendations when all the treatment options are available are illustrated in Figure 1.

The ECMM-MSG-ERC guidelines have strongly recommended the use of isavuconazole for the treatment of mucormycosis in renally compromised patients, patients with progressive disease, and patients experiencing toxicity. Isavuconazole is tagged with moderate strength as the first-line treatment of mucormycosis.

## Conclusions

The fragile patient with comorbidities and organ impairment has special requirements when it comes to antifungal usage. Although the general principles of treatment of fungal infections remain similar, there are differences in which antifungals can be safely used in this situation.

The addition of isavuconazole in the armamentarium of Indian physicians is a step towards addressing this special need. Although voriconazole and L-AmB remain the treatments of choice in *Aspergillus* and mucormycosis, respectively, isavuconazole provides an effective alternative for the treatment of IMIs. The SECURE trial has proven it to be non-inferior to voriconazole with better safety outcomes. It is proven to have a better safety and tolerability profile as compared to other azoles. The option of oral preparation also gives isavuconazole an advantage. It has a broad-spectrum antifungal activity covering clinically significant isolates of *Aspergillus spp., *especially *A. flavus* (inherently resistant to AmB preparations possibly due to higher ergosterol levels and increased enzymatic activity of the peroxidase and superoxide dismutase, with lower lipid peroxidation) and *Mucorales*. The pharmacokinetic profile of isavuconazole does not warrant routine TDM and has comparatively fewer drug-drug interactions. It is hence not surprising to see isavuconazole favourably climb the recommendation ladder in the globally recognized guidelines and being accepted as a frontrunner in the management of invasive aspergillosis and mucormycosis.
